# Machine learning improves the prediction of febrile neutropenia in Korean inpatients undergoing chemotherapy for breast cancer

**DOI:** 10.1038/s41598-020-71927-6

**Published:** 2020-09-09

**Authors:** Bum-Joo Cho, Kyoung Min Kim, Sanchir-Erdene Bilegsaikhan, Yong Joon Suh

**Affiliations:** 1 Department of Ophthalmology, Hallym University Sacred Heart Hospital, Anyang, Korea; 2 Institute of New Frontier Research, Hallym University College of Medicine, Chuncheon, Korea; 3 Department of Biomedical Science, Hallym University, Chuncheon, Korea; 4 Department of Breast and Endocrine Surgery, Hallym University Sacred Heart Hospital, 22, Gwanpyeong-ro 170 beon-gil, Dongan-gu, Anyang, 14068 Korea

**Keywords:** Cancer, Computational biology and bioinformatics, Oncology, Risk factors

## Abstract

Febrile neutropenia (FN) is one of the most concerning complications of chemotherapy, and its prediction remains difficult. This study aimed to reveal the risk factors for and build the prediction models of FN using machine learning algorithms. Medical records of hospitalized patients who underwent chemotherapy after surgery for breast cancer between May 2002 and September 2018 were selectively reviewed for development of models. Demographic, clinical, pathological, and therapeutic data were analyzed to identify risk factors for FN. Using machine learning algorithms, prediction models were developed and evaluated for performance. Of 933 selected inpatients with a mean age of 51.8 ± 10.7 years, FN developed in 409 (43.8%) patients. There was a significant difference in FN incidence according to age, staging, taxane-based regimen, and blood count 5 days after chemotherapy. The area under the curve (AUC) built based on these findings was 0.870 on the basis of logistic regression. The AUC improved by machine learning was 0.908. Machine learning improves the prediction of FN in patients undergoing chemotherapy for breast cancer compared to the conventional statistical model. In these high-risk patients, primary prophylaxis with granulocyte colony-stimulating factor could be considered.

## Introduction

Chemotherapy-induced febrile neutropenia (FN) is one of the most concerning complications in patients with breast cancer undergoing chemotherapy^1^. Neutropenia is a principal dose-limiting toxicity of myelosuppressive chemotherapy that predisposes patients to grave infections^2^. Moreover, infection in patients with neutropenia is the direct consequence of chemotherapy-induced neutropenia^3^. Chemotherapy-induced neutropenia is a principal risk factor for infection-related morbidity^4^. Further, mortality rates related with FN vary from 2 to 21%^5,6^.

Chemotherapy-induced FN commonly occurs during the initial cycle of cytotoxic therapy and increases in frequency with both duration and depth of the neutropenia^3^. In addition to an influence on quality of life, chemotherapy-induced FN exposes patients with cancer to life-threatening infections. Considering the severity of FN, the most patients who develop FN are hospitalized for evaluation and injected with broad-spectrum antibiotics. Along with infections, chemotherapy-induced FN frequently results in dose reductions and treatment delays which have been known to compromise treatment^1,7^. The risk of developing FN appears to depend on diverse factors, including patient-related factors, tumor burden, and chemotherapy regimen^6^.

Thus, primary prevention, through the administration of granulocyte colony-stimulating factor (G-CSF), is recommended by guidelines when a significant risk of FN exists^4,8,9^. G-CSF stimulates the maturation, proliferation, and release of neutrophils, leading to a dose-dependent increase in circulating neutrophils^10^. Primary prophylaxis with G-CSF decreased the risk of FN by 50% in patients with solid tumors without altering tumor response, overall survival, or infection-related mortality^8^. Currently, the criteria for the use of G-CSF and other means to reduce the risk of FN are based on low-quality evidence^11^.

Trials to prevent FN events during chemotherapy administration require an evaluation of risk factors related with the development of critical neutropenia^12^. However, this evaluation remains inaccurate^3^. Until now, no available prediction model has gained general acceptance^12^. A particularly important field of uncertainty that is emphasized by these findings is the absence of risk prediction models that estimate the risk of FN in patients reliably^8^.

Machine learning techniques have been widely adopted for the investigation of biomedical big data over the past years^13^. Recently, machine learning frameworks known as deep learning, which are based on artificial neural networks, have attracted more attention because of its notable success in predicting clinical outcomes of interest^14^. In this study, we aimed to unravel the predictive factors for and improve the prediction of FN by machine learning.

## Results

Of the 933 patients, the mean age was 51.8 ± 10.7 years. 611 (65.5%) patients underwent breast-conserving surgery. Regarding staging, 737 (79.0%) patients were staged as I/II, while 196 (21.0%) patients were staged as III/IV. The median length of follow-up was 4.9 ± 2.9 years. FN developed in 409 (43.8%) patients, and the period until the development of FN was 10.2 ± 2.8 days.

In the training dataset, 843 patients were grouped according to the presence of FN. Patients with and without FN are compared in Table 1. There was a significant difference in the incidence of FN according to age, staging, and taxane-based regimen. The group with FN was older, had advanced disease, and received taxane-based regimens more frequently. Differences between the FN and non-FN groups were also found in complete blood count/differential blood count 5 days after chemotherapy. Lymphocyte count was significantly lower in the group with FN. We calculated and validated this predictive model using the testing dataset. The demographic characteristic of the 90 patients in the testing dataset are presented in Table 2. The highest AUC value was 0.870 on the basis of logistic regression.

**Table 1:** Clinical demographic characteristics of patients with and without febrile neutropenia in the training dataset.

Parameters	FN group (n = 366)	non-FN group (n = 477)	*p* value
Age (years), means ± SD			0.004
≤ 50	157 (42.9)	276 (57.9)	< 0.001
> 50	209 (57.1)	201 (42.1)	
Body surface area (m^2^), means ± SD	1.58 ± 0.14	1.57 ± 0.13	0.498
Hypertension, n (%)	112 (30.6)	113 (23.7)	0.028
Diabetes mellitus, n (%)	42 (11.5)	44 (9.2)	0.303
Tuberculosis, n (%)	9 (2.5)	14 (2.9)	0.832
Breast-conserving surgery, n (%)	201 (55.1)	345 (72.3)	< 0.001
Tumor size (cm), mean ± SD	2.7 ± 2.0	2.3 ± 1.4	0.002
Positive lymph node, means ± SD	2.6 ± 5.2	1.2 ± 3.4	< 0.001
ER, n (%)	268 (74.0)	312 (66.0)	0.012
PR, n (%)	225 (62.2)	280 (59.2)	0.392
Her-2, n (%)	108 (29.5)	133 (27.9)	0.645
CA 15–3, means ± SD	56.3 ± 202.0	24.0 ± 136.5	0.009
TNM staging, n (%)			< 0.001
I/II	248 (67.8)	410 (86.0)	
III/IV	118 (32.2)	67 (14.0)	
Taxane-based regimen, n (%)	245 (66.9)	184 (38.6)	< 0.001
**CBC (before chemotherapy), means ± SD**			
Hemoglobin (g/dL)	12.9 ± 1.2	13.0 ± 1.4	0.426
Platelet (× 10^3^/µL)	262 ± 63	269 ± 65	0.168
Neutrophil (× 10^3^/µL)	3.725 ± 1.550	3.815 ± 1.420	0.383
Lymphocyte (× 10^3^/µL)	1.905 ± 0.603	1.971 ± 0.621	0.119
**CBC (5 days after chemotherapy), means ± SD**			
Hemoglobin (g/dL)	10.9 ± 1.7	11.5 ± 1.1	< 0.001
Platelet (× 10^3^/µL)	221 ± 82	226 ± 62	0.378
Neutrophil (× 10^3^/µL)	3.329 ± 2.278	3.067 ± 1.343	0.052
Lymphocyte (× 10^3^/µL)	0.867 ± 0.374	1.561 ± 0.593	< 0.001
SERM, n (%)	139 (38.0)	221 (46.3)	0.017
LHRH, n (%)	112 (30.6)	190 (39.8)	0.006
Aromatase inhibitor, n (%)	148 (40.4)	121 (25.4)	< 0.001
Radiation treatment, n (%)	292 (79.8)	401 (84.1)	0.122
Herceptin, n (%)	101 (27.6)	130 (27.3)	0.938

*FN* febrile neutropenia, *SD* standard deviation, *BSA* body surface area, *ER* estrogen receptor, *PR* progesterone receptor, *Her-2* human epidermal growth factor receptor 2, *CA* cancer antigen, *CBC* complete blood count, *SERM* selective estrogen receptor modulator, *LHRH* luteinizing hormone-releasing hormone, *F/U* follow-up.

**Table 2:** Clinical demographic characteristics of patients with and without febrile neutropenia in the testing dataset.

Parameters	FN group (n = 43)	non-FN group (n = 47)	*p* value
Age (years), means ± SD			0.050
≤ 50	13 (30.2)	24 (51.1)	
> 50	30 (69.8)	23 (48.9)	
Body surface area (m^2^), means ± SD	1.61 ± 0.14	1.63 ± 0.14	0.383
Hypertension, n (%)	12 (27.9)	11 (23.4)	0.638
Diabetes mellitus, n (%)	5 (11.6)	5 (10.6)	1.000
Tuberculosis, n (%)	0 (0)	0 (0)	1.000
Breast-conserving surgery, n (%)	28 (65.1)	37 (78.7)	0.166
Tumor size (cm), mean ± SD	2.7 ± 1.3	2.5 ± 1.2	0.490
Positive lymph node, means ± SD	1.7 ± 4.0	0.3 ± 1.4	0.038
ER, n (%)	34 (79.1)	26 (55.3)	0.046
PR, n (%)	26 (60.5)	18 (38.3)	0.057
Her-2, n (%)	12 (27.9)	7 (14.9)	0.196
CA 15–3, means ± SD	15.2 ± 12.0	11.7 ± 7.8	0.140
TNM staging, n (%)			0.003
I/II	33 (76.7)	46 (97.9)	
III/IV	10 (23.3)	1 (2.1)	
Taxane-based regimen, n (%)	23 (53.5)	14 (29.8)	0.032
**CBC (before chemotherapy), means ± SD**			
Hemoglobin (g/dL)	13.0 ± 1.6	13.1 ± 1.2	0.940
Platelet (× 10^3^/µL)	263 ± 56	265 ± 77	0.590
Neutrophil (× 10^3^/µL)	3.661 ± 1.574	4.006 ± 1.683	0.550
Lymphocyte (× 10^3^/µL)	2.024 ± 0.575	2.193 ± 0.640	0.250
**CBC (5 days after chemotherapy), means ± SD**			
Hemoglobin (g/dL)	10.7 ± 1.1	5.314 ± 1.593	0.017
Platelet (× 10^3^/µL)	219 ± 99	11.3 ± 1.0	0.170
Neutrophil (× 10^3^/µL)	3.977 ± 3.468	3.348 ± 1.208	0.220
Lymphocyte (× 10^3^/µL)	0.838 ± 0.339	1.703 ± 0.683	< 0.001
SERM, n (%)	13 (30.2)	23 (48.9)	0.087
LHRH, n (%)	11 (25.6)	20 (42.6)	0.121
Aromatase inhibitor, n (%)	22 (51.2)	14 (29.8)	0.053
Radiation treatment, n (%)	35 (81.4)	41 (87.2)	0.564
Herceptin, n (%)	13 (30.2)	10 (21.3)	0.346

*FN* febrile neutropenia, *SD* standard deviation, *ER* estrogen receptor, *PR* progesterone receptor, *Her-2* human epidermal growth factor receptor 2, *CA* cancer antigen, *CBC* complete blood count, *SERM* selective estrogen receptor modulator, *AI* aromatase inhibitor, *LHRH* luteinizing hormone-releasing hormone, *F/U* follow-up.

**Figure 1:**
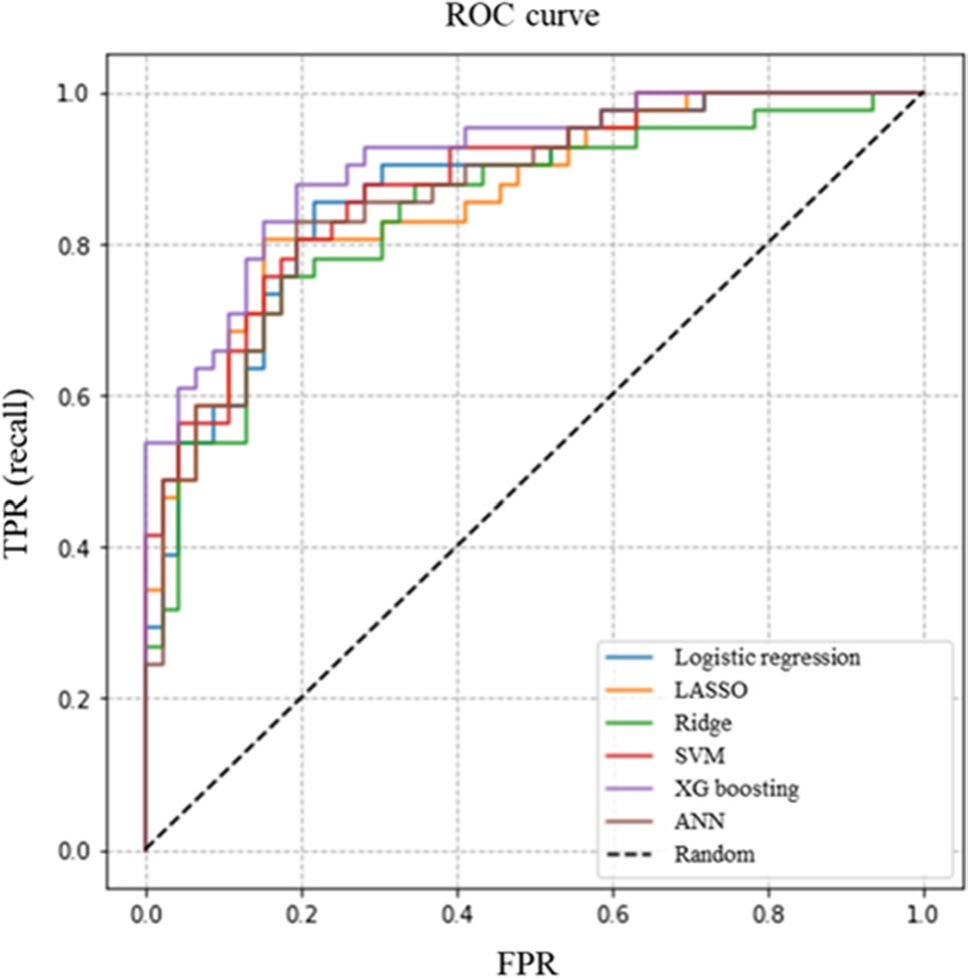
The AUC of each algorithm shown using colored lines. The image was drawn in Python 3.6. *AUC* area under the curve, *ROC* receiver operating characteristic, *TPR* true positive rate, *LASSO* least absolute shrinkage and selection operator regression, *SVM* support vector machine, *ANN* artificial neutral network.

**Figure 2:**
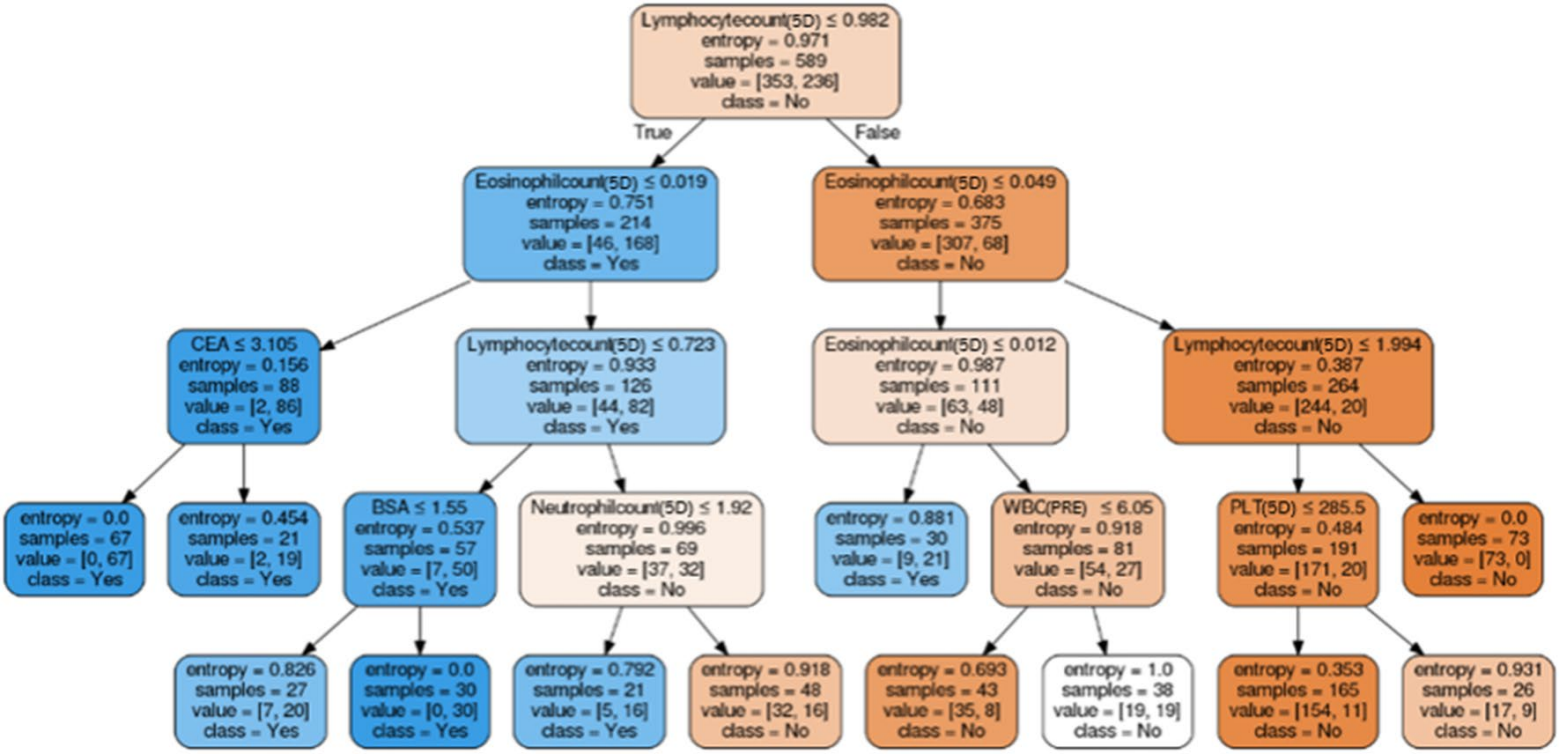
Detailed cut-off values displayed in a decision tree model. The image was drawn in Python 3.6. *5D* 5 days after chemotherapy, *CEA* carcinoembryonic antigen, *BSA* body surface area, *WBC* white blood cell, *PRE* pretreatment, *PLT* platelet.

Factors associated with FN were selected by machine learning algorithms. The performances of prediction models in the testing dataset are presented in Table 3. XGboosting showed the best performance with an AUC of 0.908. The AUC of each algorithm is presented in Fig. 1. Data collected for hierarchical levels were used as input data for the decision tree model. The root node of the decision tree was lymphocyte count 5 days after chemotherapy, and the cut-off was 0.982 (× 10^3^/µL) (Fig. 2).

**Table 3:** Performance of machine learning algorithms for the prediction of febrile neutropenia.

	LR	DT	XGboosting	LASSO	SVM	ANN
AUC	0.870	0.855	0.908	0.862	0.880	0.865
Accuracy	0.781	0.759	0.816	0.805	0.782	0.782
Sensitivity	0.878	0.707	0.829	0.805	0.829	0.854
Specificity	0.696	0.804	0.804	0.804	0.739	0.717
PPV	0.720	0.763	0.791	0.786	0.739	0.729
NPV	0.865	0.755	0.841	0.822	0.829	0.846

*LR* logistic regression, *DT* decision tree, *LASSO* least absolute shrinkage and selection operator, *SVM* support vector machine, *ANN* artificial neural network, *AUC* area under the curve, *PPV* positive predictive value, *NPV* negative predictive value.

## Discussion

In the present study, multivariate analysis demonstrated predictive factors for FN, including age, staging, and taxane-based regimen. The lymphocyte count 5 days after chemotherapy was also a strong predictive factor for FN. Based on these findings, logistic regression showed an AUC of 0.870 for validation. Even in machine learning, the lymphocyte count 5 days after chemotherapy was the strongest predictive factor for FN. The AUC improved by machine learning was 0.908, although with a slight difference.

Chemotherapy regimen is one of the main determinants of the risk of FN as shown in the present study. In practice, some regimens are more myelotoxic than others^4^. Taxane- and anthracycline-based regimens were previously reported as regimens with a high risk of FN when used for the treatment of breast cancer^4^. CMF is less toxic than AC or FA(E)C^3^. Because the rates of FN for these and similar regimens vary considerably, it is difficult to determine the actual risk^15^. In addition to the regimen-specific risks, evaluating the individual risk factors in each patient can be valuable in determining appropriate treatment^16^.

The cycle number of the current round of chemotherapy is an important factor for FN, although only the first cycle was investigated in the present study^11^. Previous studies have demonstrated that the first cycle of chemotherapy is related with a greater risk for the development of FN than subsequent cycles^2,17^. The decreased risk of FN after subsequent cycles may be the result of clinicians’ understanding of the nadir of blood counts and clinical features of patients during the first round of chemotherapy. The history of FN in a patient is a generally recognized risk factor for the development of FN^11,18^. Guidelines recommend the use of G-CSF as secondary prophylaxis in patients who develop FN during the equitoxic chemotherapy regimen, considering the patient’s prior tolerance to chemotherapy^11^.

Blood counts may indicate comorbid conditions, the extent of disease, or individual response to cytotoxic chemotherapy. Even in the present study, the lymphocyte count 5 days after chemotherapy was the strongest predictive factor for FN. The slow decrease of the nadir of the lymphocyte count is apparently protective against FN^19^. Higher lymphocyte counts 5 days after chemotherapy may reflect higher resistance to infection, as these patients may have the potential to activate their cellular or humoral immunity rapidly^19–21^. However, the explicit role of lymphocytes in the development of FN remains to be elucidated.

Previous studies have reported that prophylactically administered G-CSF is significantly related with a lower risk of FN^10,22^. Primary prophylaxis with G-CSF can decrease the need for dose delay or reduction, antibiotics, and hospital admission^4,18^. Moreover, prophylactic G-CSF reduces early death, including infection-related mortality^10,22^. Currently, guidelines recommend prophylaxis with G-CSF when the FN risk is high (> 20%) on the basis of either chemotherapy regimen alone (high-risk regimen) or the combination of chemotherapy regimen (intermediate-risk regimen with 10–20% FN risk) and personal risk factors^4,9^.

In this study, some machine learning algorithms outperformed logistic regression. This phenomenon has been observed in many prediction models using machine learning^13,14^. Logistic regression models are an extension of linear models using logit function as a link. Therefore, a non-linear interaction between associated factors and the outcome may not be fitted optimally. Using non-linear functions, machine learning recognizes the patterns present in the medical data and predicts the outcomes by minimizing the error^23^.

Our machine learning algorithm can be implemented in a clinical workflow to bridge the gap between research and practice. Considering that the period until the development of FN was 10.2 ± 2.8 days in our study, clinicians may use parameters including the complete blood count/differential blood count 5 days after chemotherapy to decide whether to use prophylactic G-CSF. Therefore, we envision a software tool for the prediction of FN after chemotherapy in patients with breast cancer (Supplementary Fig. S1). The software provides the predicted probability of FN if parameters regarding FN are entered using a user-friendly interface.

To the best of our knowledge, this study is the first to improve the prediction of FN after chemotherapy in patients with breast cancer by machine learning. Our predictive model defines the risk of FN after chemotherapy. The current model represents progress in predicting FN and optimizing protection against its development. This machine learning model has the potential to become a routine tool in daily clinical practice to guide the use of prophylactic G-CSF.

The present study has some limitations. First, our data showed the high rate of FN, considering that the relevant literature reported the incidence of FN as 10–50%^8,9,24–28^. In the current study, inpatients were purely selected because they had more lucid serial data. However, hospitalized patients usually have more severe status rather than outpatients, which can cause a selection bias. Moreover, according to the criteria of our national health insurance coverage G-CSF should be given at less than 500 of neutrophils or at less than 1,000 of neutrophils if patients have fever. Therefore, generalization should be avoided. Second, only the first cycle of each regimen was investigated. Subsequent cycles were not regarded as independent since FN may be affected by the accumulation of drugs during previous cycles. Thus, a more customized model needs to be developed for the subsequent cycles. Lastly, the decision to use G-CSF was not analyzed. Regarding the use of G-CSF, cost and national insurance coverage should be considered jointly.

In conclusion, machine learning improved the prediction of FN in patients undergoing chemotherapy for breast cancer. In these high-risk patients, primary prophylaxis with G-CSF could be considered. With this strategy, patient safety could be ensured during chemotherapy in patients with breast cancer.

## Methods

### Study design

**Figure 3:**
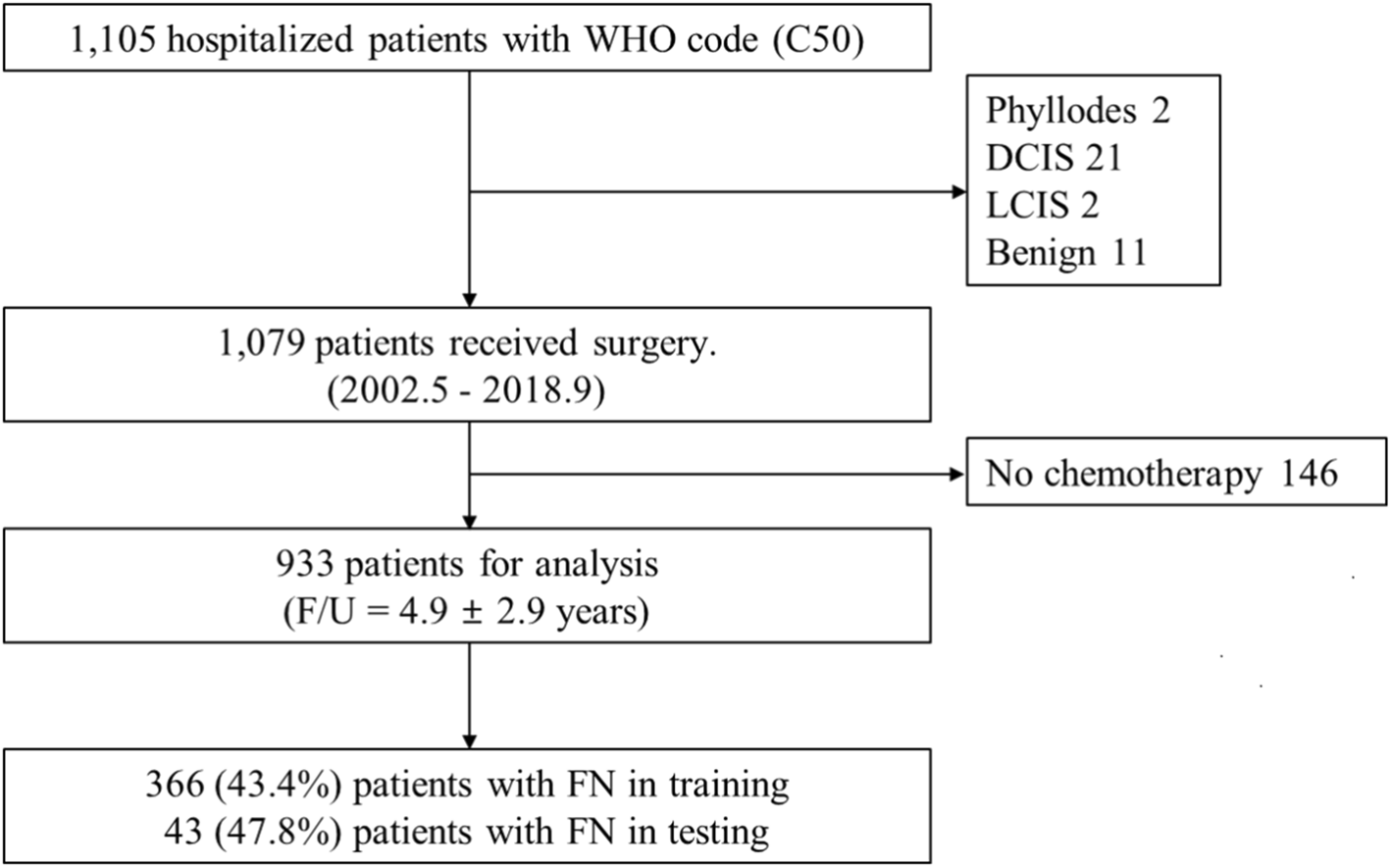
Flow diagram depicting the study design. The image was drawn in Microsoft PowerPoint 2016. *WHO* World Health Organization, *DCIS* ductal carcinoma in situ, *LCIS* lobular carcinoma in situ, *F/U* follow-up, *FN* febrile neutropenia.

Medical records of 1,105 hospitalized patients diagnosed with breast cancer between May 2002 and September 2018 in the Department of Breast and Endocrine surgery, Hallym University Sacred Heart Hospital were selectively reviewed for inclusion. Among them, 1,079 patients underwent surgery and were confirmed pathologically as having breast cancer. Finally, of the 1,079 patients, 933 who received chemotherapy after surgery were included in this study (Fig. 3). All patients received the first cycle of full-dose chemotherapy in the hospital, and biometric data were recorded during the treatment period. This study was approved by the Institutional Review Board of Hallym University Sacred Heart Hospital (No. 2018-04-018) and adhered to the tenets of the Helsinki Declaration. The requirement for written informed consent was waived by the Institutional Review Board.

### Datasets

The entire cohort was divided into a training and testing dataset which were mutually exclusive. The training dataset was built with 843 patients treated between May 2002 and January 2018. The testing dataset consisted of 90 patients treated between February 2018 and September 2018 and was used to validate the performance of machine learning models. In both datasets, patients who had any missing data for clinical, pathological, or therapeutic variables of interest were excluded from the analyses.

### Assessments

Demographic, clinical, pathological, and therapeutic information were obtained from the medical records of study participants. Tumors were staged according to the 8th edition of the American Joint Committee on Cancer staging system. FN was defined as the incidence of fever of 38.3 °C or 38.0 °C for over 1 h orally, and neutrophil count < 500 or 500–999/mm^3^ with predicted drop to < 500/mm^3^ over next 48 h^9^. During the first cycle of chemotherapy, each patient was monitored carefully for the development of FN.

### Analysis

To extract the factors associated with FN, classical and recent machine learning algorithms were applied. Least absolute shrinkage and selection operator regression, ridge regression, support vector machine, decision tree, XGboosting, and artificial neural network were used for machine learning algorithms. Conventional stepwise logistic regression was used as a reference method. Factors associated with FN were selected from the dataset using the recursive feature elimination method^29^. The *p* value used to select and remove the factor in the forward stepwise process was 0.05. Factor selection and model construction were done on the platform with scikit-learn 0.20 in Python 3.6 (Python Software Foundation, Wilmington, DE). Prediction models were constructed for each machine learning algorithm with the training dataset using the optimal feature subset for each machine-learning algorithm. Five-fold cross-validation was used for evaluation. The performance of the prediction models was evaluated in the testing dataset. The area under the curve (AUC) was used as the main measurement.

## Supplementary information

Supplementary file1

Supplementary file2

## Data Availability

All the data supporting the findings of this study are available from the corresponding author upon reasonable request.
