# Supplementary figures and images for: Machine learning improves the prediction of febrile neutropenia in Korean inpatients undergoing chemotherapy for breast cancer
Source: Sci Rep. 2020 Sep 9;10:14803. doi: 10.1038/s41598-020-71927-6 (PMC7481240; doi:10.1038/s41598-020-71927-6)

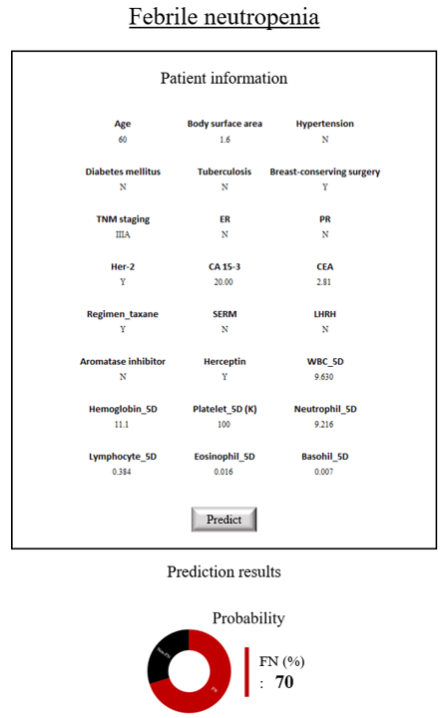

Supplement: Supplementary file 1 — Supplementary file1 [file 41598_2020_71927_MOESM1_ESM.tif]
