# Supplementary material for: Machine learning improves the prediction of febrile neutropenia in Korean inpatients undergoing chemotherapy for breast cancer
Source: Sci Rep. 2020 Sep 9;10:14803. doi: 10.1038/s41598-020-71927-6 (PMC7481240; doi:10.1038/s41598-020-71927-6)
Supplement: Supplementary file 2 — Supplementary file2 [file 41598_2020_71927_MOESM2_ESM.docx]

**Journal Name: Scientific Reports**

**Machine learning improves the prediction of febrile neutropenia in Korean inpatients undergoing chemotherapy for breast cancer**

Bum-Joo Cho^1^, Kyoung Min Kim^2^, Sanchir-Erdene Bilegsaikhan^3^, Yong Joon Suh^4*^

^1^Department of Ophthalmology, Hallym University Sacred Heart Hospital, Anyang, Korea

^2^Institute of New Frontier Research, Hallym University College of Medicine, Chuncheon, Korea

^3^Department of Biomedical Science, Hallym University, Chuncheon, Korea

^4^Department of Breast and Endocrine Surgery, Hallym University Sacred Heart Hospital, Anyang, Korea

**^*^Corresponding Author:**

Yong Joon Suh, MD, PhD

Department of Breast and Endocrine Surgery

Hallym University Sacred Heart Hospital

22, Gwanpyeong-ro 170 beon-gil, Dongan-gu, Anyang, 14068, Korea

Tel: +82-31-381-7698, Fax: +82-31-380-5932

E-mail: nicizm@gmail.com

**Supplementary Figure Legend**

**Supplementary Figure S1.** Envisioned software tool for the prediction of FN after chemotherapy in patients with breast cancer. The image was drawn in Microsoft PowerPoint 2016. *FN*, febrile neutropenia
